# Candesartan-Associated Lichenoid Drug Eruption

**DOI:** 10.7759/cureus.85920

**Published:** 2025-06-13

**Authors:** Kazuyoshi Iijima, Yoshihito Mima

**Affiliations:** 1 Department of Dermatology, Teikyo Mizonokuchi Hospital, Kanagawa, JPN; 2 Department of Dermatology, Tokyo Metropolitan Police Hospital, Tokyo, JPN

**Keywords:** antihypertensive agents, candesartan, cutaneous adverse drug reactions (cadrs), lichenoid drug eruption, skin lesions

## Abstract

Lichenoid drug eruption (LDE) is an uncommon adverse drug reaction, with few published cases implicating the angiotensin II receptor blocker candesartan. We report a 67-year-old woman who developed pruritic violaceous papules and plaques on the trunk and extremities two years after initiating candesartan. Skin biopsy confirmed LDE. The eruption gradually resolved over five to six months after candesartan was replaced by amlodipine, with no recurrence observed at the 12-month follow-up. This case underscores the diagnostic challenges associated with LDE: latency may extend over years, clinical and histopathologic features closely mimic idiopathic lichen planus, and confirmatory tests, such as patch testing and drug-induced lymphocyte stimulation testing, often yield low sensitivity. Dermatologists and prescribing physicians should maintain a high index of suspicion for drug-induced lichenoid reactions, even when no recent medication changes are reported, as prompt recognition and withdrawal of the offending agent are essential for achieving durable remission.

## Introduction

Cutaneous adverse drug reactions (CADRs) account for approximately 2-3% of all adverse drug events [1] and represent one of the most common reasons for dermatology consultation. Among these, cutaneous lichenoid drug eruption (LDE) is distinctly uncommon, comprising only 0.4-2.8% of reported CADRs [1]. Clinically, LDE manifests as pruritic, violaceous papules and plaques, often symmetrically distributed across the trunk and extremities, closely resembling idiopathic lichen planus (LP) [2].

Differentiating LDE from LP remains challenging, as the clinical presentation and histopathological features substantially overlap. However, subtle histologic clues, such as the presence of eosinophils, focal parakeratosis, or deeper perivascular inflammation, may favor a drug-induced etiology [3]. Thus, while histological evaluation is essential, a meticulous review of the patient’s medication history remains critical for accurate diagnosis.

The diagnostic challenge is further compounded by the characteristically prolonged latency of LDE. A recent review aggregating 323 published cases reported a mean latency of 15.7 weeks (range: 0.1-208 weeks) from drug initiation to eruption onset, and a median of 14.2 weeks (range: 0.7-416 weeks) to complete resolution after drug discontinuation [4]. Over 100 medications have been implicated in LDE, including non-steroidal anti-inflammatory drugs (NSAIDs), beta-blockers, antimalarials, antihypertensive agents, and immune checkpoint inhibitors (ICIs), with ICIs now accounting for more than 40% of newly reported cases [4].

Among cardiovascular agents, angiotensin II receptor blockers (ARBs) are widely prescribed for hypertension, heart failure, and diabetic nephropathy. Although generally well tolerated, ARBs have occasionally been associated with cutaneous adverse reactions, most commonly exanthematous eruptions [5]. LDE is much less frequently reported, with approximately a dozen cases described to date. Most of cases have involved losartan or valsartan, whereas reports implicating candesartan remain particularly rare; only a few such cases have been documented in the literature [6,7].

Because ARBs are often initiated and renewed in primary care or internal medicine settings, mild or nonspecific eruptions may be misattributed to other dermatosis, particularly when the latency exceeds several months. If the causative agent is not discontinued, lesions may persist or relapse for prolonged periods despite topical corticosteroid therapy. Consequently, LDE remains underrecognized, and its true incidence is likely underestimated.

We report a case of candesartan-induced LDE that exemplifies these diagnostic challenges and highlights the need for heightened interdisciplinary vigilance to achieve prompt identification and durable remission.

## Case presentation

A 67-year-old woman presented with a one-year history of intermittently relapsing pruritic eruptions. The lesions first appeared approximately two years after she had initiated candesartan (4 mg/day) for the treatment of hypertension and progressively involved the trunk and extremities. She was otherwise healthy, was not taking any other medications, and reported no recent use of topical products, herbal supplements, or international travel. 

**Figure 1:**
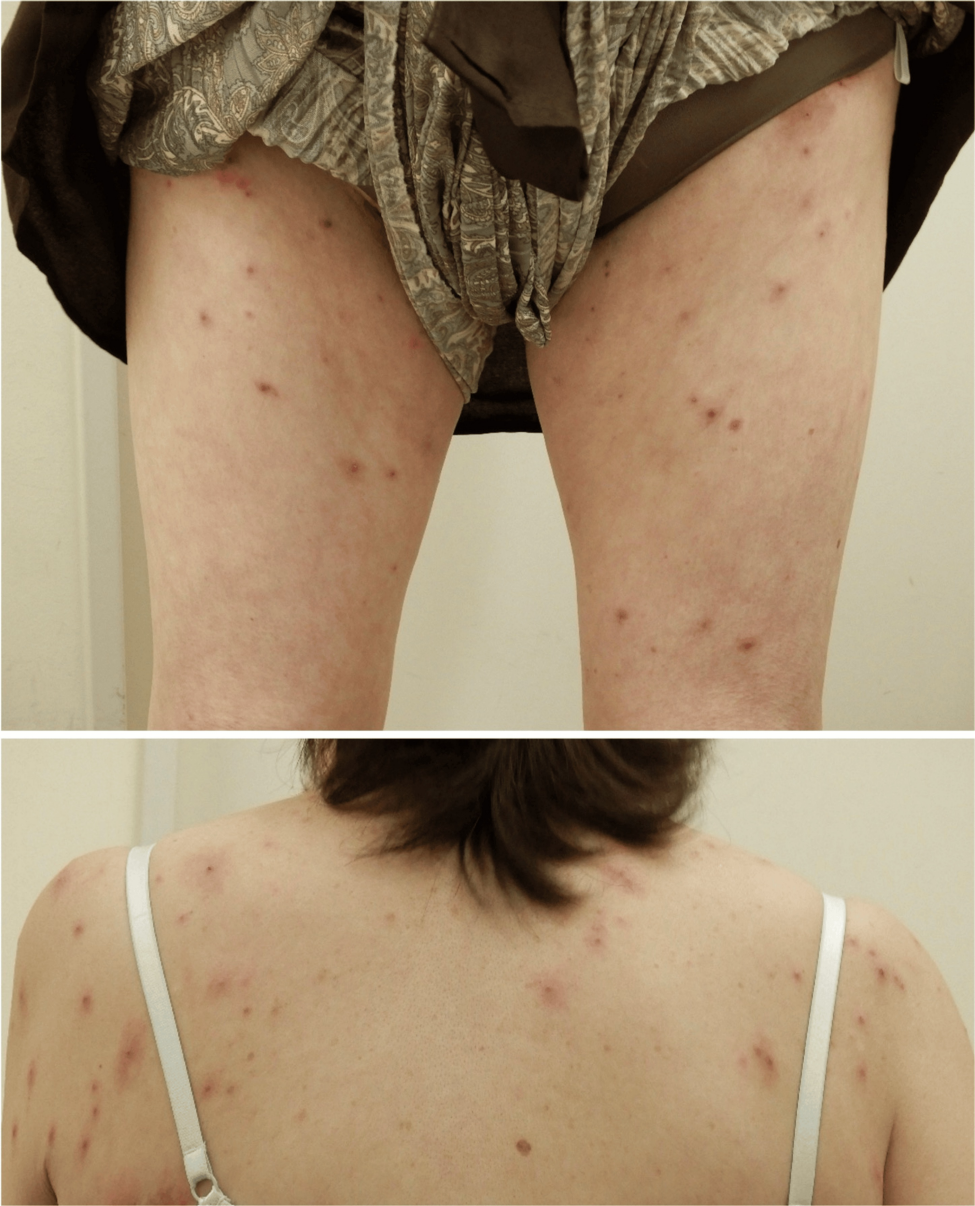
Multiple erythematous maculopapular eruptions were symmetrically distributed over the trunk and extremities.

On physical examination, multiple erythematous maculopapular eruptions were symmetrically distributed over the trunk and extremities (Figure 1).

No mucosal, scalp, or nail abnormalities were observed. Laboratory investigations, including complete blood count, liver and renal function panels, thyroid function tests, hepatitis B and C serologies, HIV antibody testing, and antinuclear antibody screening, were all within normal limits.

**Figure 2:**
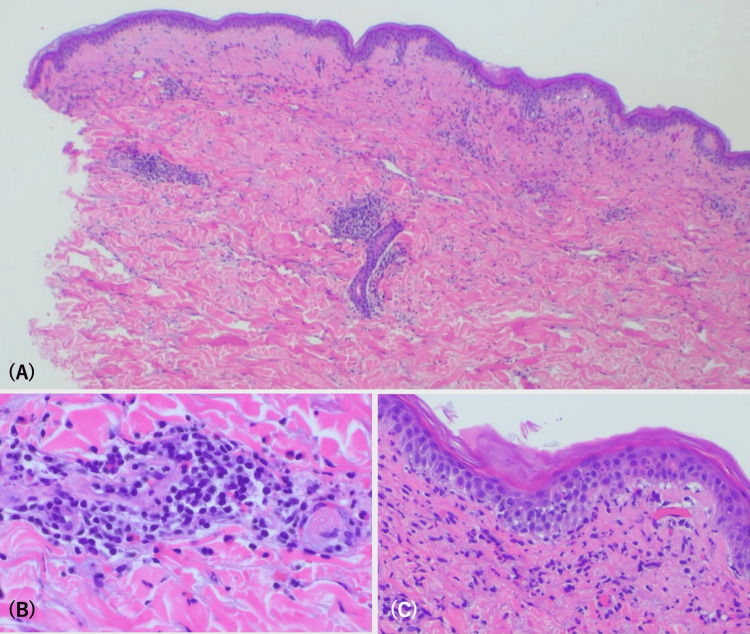
Histopathological findings. (A) Irregular acanthosis with a saw-toothed appearance of the rete ridges, wedge-shaped hypergranulosis, and a band-like lymphocytic infiltrate at the dermoepidermal junction. (B) Inflammatory cell infiltration surrounding dermal capillaries. (C) Basal vacuolar alteration is consistent with interface dermatitis.

A punch biopsy from a lesion on the forearm demonstrated focal hypergranulosis, irregular acanthosis with an early sawtooth pattern of rete ridges, vacuolar alteration of the basal cell layer, a relatively sparse band-like lymphocytic infiltrate, and perivascular inflammation. Several eosinophils were also noted in the superficial dermis (Figure 2). These findings were compatible with LDE rather than LP (Figure 2).

In light of the clinical morphology, chronic relapsing course, histopathological features, and the absence of other likely causes, the diagnosis of candesartan-induced LDE was considered. The utility of confirmatory tests, such as patch testing and drug-induced lymphocyte stimulation testing (DLST), was discussed; however, the patient declined further testing after being informed of their limited diagnostic sensitivity in LDE.

**Figure 3:**
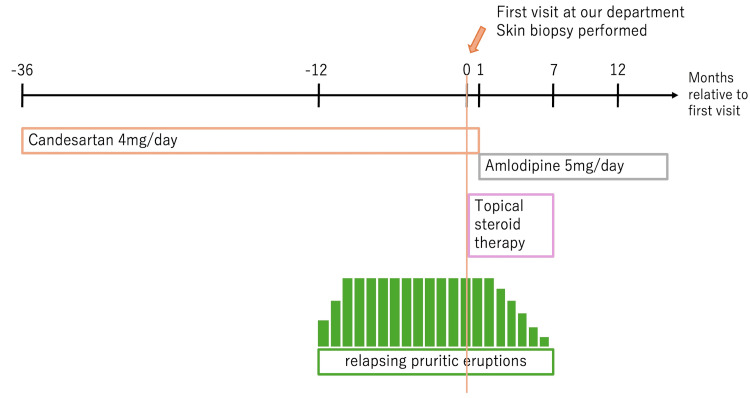
Timeline of clinical events in this case. Pruritic eruptions began approximately two years after the initiation of candesartan and persisted despite topical steroid therapy. Following the switch to amlodipine, the lesions gradually improved and resolved completely in six months.

Topical corticosteroid therapy with betamethasone valerate 0.05% ointment, initiated at the time of initial presentation and continued throughout the course, resulted in only partial symptomatic relief. Following consultation with her primary care physician, candesartan was discontinued and replaced with amlodipine 5 mg daily. Gradual symptomatic improvement was observed, with decreasing pruritus and flattening of the papules, leaving only faint post-inflammatory hyperpigmentation. The complete clinical resolution was achieved after six months. At 12-month follow-up, no recurrence was noted, and blood pressure remained well controlled with amlodipine monotherapy. Based on the temporal association with drug exposure, histopathological findings, and resolution after drug withdrawal, a final diagnosis of candesartan-induced LDE was established. A timeline summarizing the clinical course is shown in Figure 3.

## Discussion

LDE and LP are clinicopathologic mimics, sharing key histopathological features, such as orthohyperkeratosis, focal wedge-shaped hypergranulosis, vacuolar alteration of the basal cell layer, and a band-like lymphocytic infiltrate at the dermoepidermal junction [2]. However, several subtle histopathologic features favor a diagnosis of LDE over LP. The presence of eosinophilic infiltrates, focal parakeratosis, focal disruption of the granular layer, Civatte bodies in the stratum corneum and granulosum, and extension of lymphocytic inflammation around superficial and deep dermal vessels have been reported as clues suggestive of drug-induced etiology [8,9]. In our case, all of these features were observed, supporting a drug-induced rather than idiopathic etiology.

In addition to idiopathic lichen planus, lichen planus-like drug eruption (LDE) should be differentiated from other mimickers, such as maculopapular drug eruption, psoriasis, and early-stage cutaneous T-cell lymphoma (CTCL). Maculopapular drug eruptions usually appear abruptly and resolve rapidly following drug withdrawal [5]. Psoriasis may resemble LDE clinically but typically exhibits regular acanthosis and Munro microabscesses. Early CTCL can mimic LDE both clinically and histologically. Although epidermotropism of atypical lymphocytes is a key feature, it may be subtle or absent in some cases [10]. Conversely, wedge-shaped hypergranulosis and Civatte bodies, which are typically observed in LDE, are generally not seen in CTCL [8,9].

Causative agents have expanded dramatically over the past decade. More than 100 different drugs have been implicated in LDE, with ICIs accounted for 42% of reported cases, followed by tyrosine kinase inhibitors (12%) and anti-tumor necrosis factor-alpha (TNF-α) antibodies (4%) [4]. Renin-angiotensin-aldosterone system (RAAS) inhibitors accounted for 3.7% of cases [4], with candesartan involvement reported only sporadically [6,7].

The latency between drug initiation and the onset of LDE is typically prolonged. Maul et al. reported an average latency of 15.7 weeks (approximately 3.6 months), with a wide range from a few days to up to four years [4]. This variability is thought to be influenced by multiple factors, including drug dosage, prior sensitization, concomitant medications, and host immune predisposition [7]. For antihypertensive agents such as ARBs, a separate report proposed a longer mean latency of about seven months (range: 4-12 months) [9], which still falls within Maul’s overall range. In our case, the latency period from drug initiation to the onset of skin lesions was 24 months, which falls within the reported range for LDE. No other potential triggers, such as newly introduced medications, infections, or systemic conditions, were identified during clinical evaluation. Resolution after drug discontinuation may also be delayed, with a median time to improvement of 14.2 weeks (approximately 3.3 months) [4]. This protracted clinical course contrasts sharply with maculopapular drug eruptions, which typically resolve within three to seven days after cessation of the offending drug [5]. Such prolonged latency, combined with delayed resolution after drug discontinuation, explains why long-standing therapies are easily overlooked during routine drug reconciliation and highlights the need for additional case aggregation for ARB-induced LDE.

Although the precise mechanisms underlying LDE remain incompletely elucidated, accumulating evidence supports the notion that it represents a CD8⁺ T cell-mediated autoimmune response targeting drug-modified basal keratinocytes [7]. Mechanistically, CD8⁺ cytotoxic T lymphocytes are believed to induce apoptosis of basal keratinocytes through both the granzyme B-perforin axis and Fas-Fas ligand interactions [3,7]. Notably, granzyme B-expressing lymphocytes have been observed in greater abundance in LDE lesions compared to LP, correlating with clustered apoptotic keratinocytes--a finding suggestive of a heightened cytotoxic response [3,7]. Additional pathways involving plasmacytoid dendritic cells and CD14/Toll-like receptor (TLR) signaling--described in the setting of ICI-associated LDE--further implicate combined innate and adaptive immune dysregulation [11].

The characteristically prolonged latency of LDE, often spanning months to years after drug initiation, may reflect gradual neoantigen formation, epitope spreading, and clonal expansion of autoreactive T cells. Furthermore, the persistence of cutaneous lesions even after withdrawal of the causative agent may be driven by long-lived tissue-resident memory T cells (TRM) within the epidermis and dermis, in conjunction with the sustained expression of adhesion molecules such as ICAM-1, which perpetuates local immune activation.

Establishing a definitive causal relationship with a specific drug remains challenging. In practice, even if a drug is re-administered, the recurrence of skin lesions may take several months, and patients are often reluctant to undergo rechallenge, making it an impractical approach in most cases. Moreover, diagnostic tools such as patch testing and drug-induced lymphocyte stimulation testing (DLST) frequently yield false-negative results [7], likely due to the T cell-mediated nature of the reaction and the possibility that drug metabolites, rather than the parent compound, act as the true antigen-a limitation that appears to be relevant in LDE. Consequently, the diagnosis of LDE typically relies on a combination of clinicopathologic concordance, a thorough review of long-term medication history, and clinical improvement following discontinuation of the suspected agent. To further support causality, structured assessment tools such as the Naranjo Adverse Drug Reaction Probability Scale may be utilized [12]. In our case, a total score of six was obtained, corresponding to a ‘probable’ adverse drug reaction. While not definitive, such tools can reinforce clinical suspicion, particularly when rechallenge is impractical and confirmatory tests yield inconclusive results. The interpretation of such scores should be made in the context of disease characteristics, including prolonged latency and delayed resolution, as previously discussed.

Because antihypertensive therapy is typically managed by internists, dermatologists must maintain a high index of suspicion for LDE and proactively advocate for drug withdrawal or substitution when suspected. Failure to identify and discontinue the culprit drug can result in years of relapsing pruritus and unnecessary treatment escalation, whereas prompt intervention, as demonstrated in this case, may lead to remission within the typical three- to six-month timeframe. Raising awareness of LDE among both dermatologists and prescribing physicians is essential to minimize diagnostic delays and improve patient outcomes.

## Conclusions

This case illustrates the diagnostic complexity of candesartan-induced lichenoid drug eruption and reinforces the need for high clinical suspicion, even in patients receiving long-term, stable medication. Prompt recognition and drug withdrawal can lead to durable remission and prevent unnecessary escalation of treatment. Routine review of long-term medications should be part of dermatologic evaluation, especially when latency is unpredictable.
